# Commentary: Targeting chemoattractant chemokine (C–C motif) ligand 2 derived from astrocytes is a promising therapeutic approach in the treatment of neuromyelitis optica spectrum disorders

**DOI:** 10.3389/fimmu.2023.1279782

**Published:** 2023-09-27

**Authors:** Jingdan Zhang, Jinbo Cheng

**Affiliations:** Center on Translational Neuroscience, College of Life & Environmental Science, Minzu University of China, Beijing, China

**Keywords:** NMOSD, neuroinflammation, CCL2, monocyte, astrocyte

## Introduction

Neuromyelitis optica spectrum disorder (NMOSD) is a highly recurrent and disabling inflammatory autoimmune disease in the central nervous system (CNS), characterized with severe optic neuritis and myelitis. It has been reported that monocytes derived from bone marrow was activated and involved in this disease. Moreover, inflammatory cytokine IL-6 levels was significantly upregulated in both serum and cerebrospinal fluid of NMO patients, and activated monocytes was one identified source of this increase (1, 2). Multiple evidences showed that microglia were local immune cells of the nervous system, and abnormal neuroinflammation caused by activation of microglia played a critical role in CNS diseases (3–5). Pathological features of NMO anatomy showed that reactive macrophages/microglia clustered in focal patterns in the tubule, ependyma, and other Aquaporin-4 (AQP4) immune response areas of the brain (6), suggesting that microglia also played an important role in NMOSD. Moreover, astrocytes were the most and largest glial cells in the CNS, and astrocytes regulated ionic homeostasis and responded to environmental factors, all of which have been implicated in neurological disorders.

**Figure 1:**
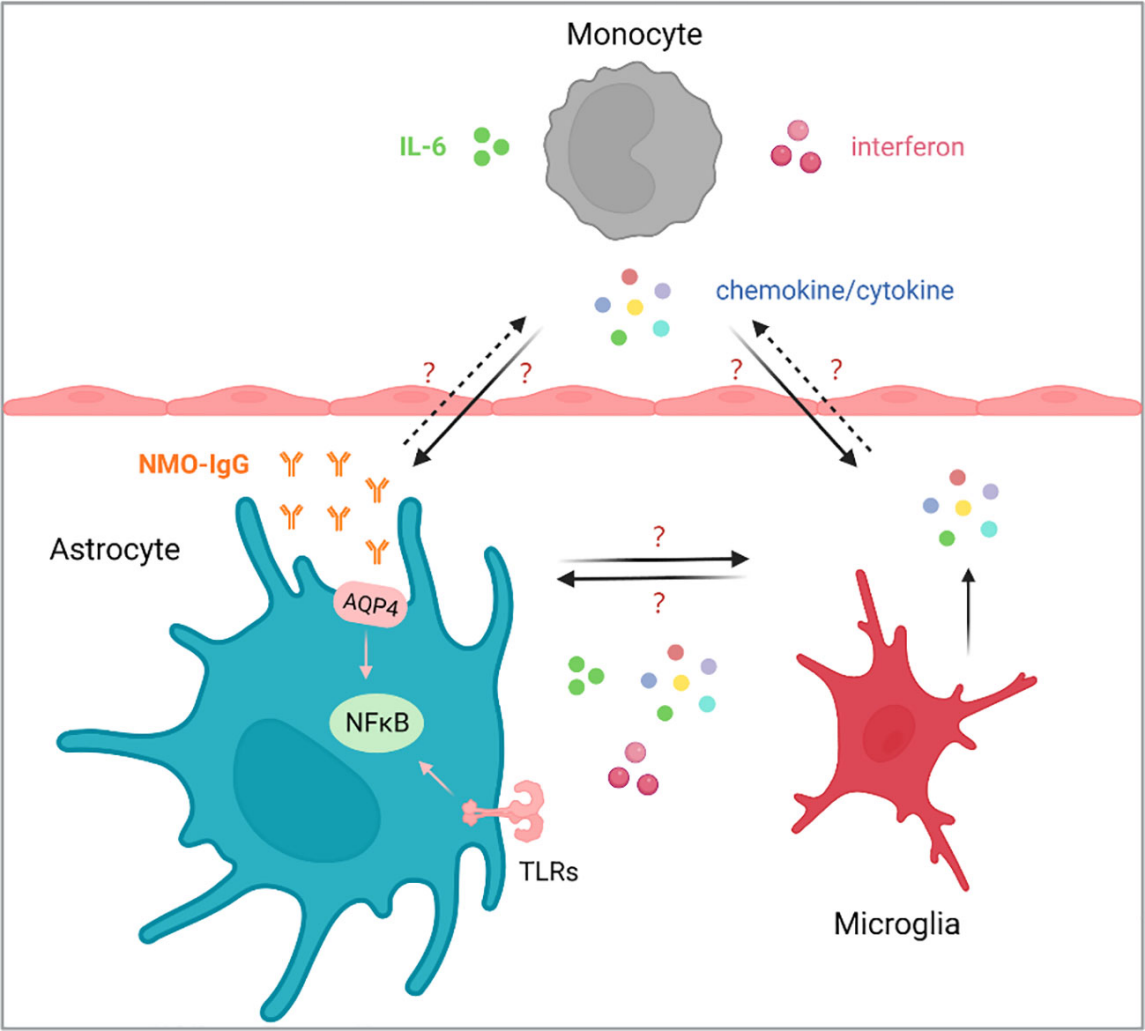
The homeostasis of monocytes, astrocytes and microglia plays an important role in the NMOSD. The occurrence and development of NMOSD are related to IL-6 and AQP4 antibodies, and blocked IL-6 has been reported to reduce the production of AQP4-IgG. How chemokines/cytokines produced by monocytes affect the function of astrocytes and microglia, and how astrocytes and microglia respond, as well as the communication between astrocytes and microglia are still largely unknown.

In 2004, Lennon et al. found an autoantibody binding to astrocyte AQP4 in the serum of NMO patients and defined it as NMO-IgG (7). It was currently believed that NMO-IgG activated the complement after binding with AQP4, leading to the destruction of the blood-brain barrier, astrocytic injury and secondary demyelination. Meanwhile, the interaction of NMO-IgG with AQP4 also triggered multiple immune-related pathways, such as chemokine/cytokine, interferon and NF-κB pathway, suggesting that the degree of NMO lesions depended on the immune microenvironment, in which multiple immune cells, including monocytes, microglia, and astrocyte, were all involved (8–10). However, the regulatory mechanism of changes of immune microenvironment in the pathogenesis of NMO lesions are largely unknown (**Figure 1**).

## The role of CCL2 in NMO-IgG-induced astrocyte injury

Recently, Wang et al. published one paper in *Frontiers in immunology*, reported a chemokine (C–C motif) ligand 2 (CCL2) and CCR2^+^-mediated positive feedback cascade loop contributed to the pathogenesis of NMO (11), providing a new insight for the treatment of NMOSD. The authors found that the expression of CCL2 in the cerebrospinal fluid were significantly upregulated in NMOSD patients compared to other non-inflammatory neurological diseases (ONDs) patients. Meanwhile, the expression of CSF-CCL2 was significantly upregulated in AQP4-IgG seropositive patients. In order to investigate the role of CCL2 in the astrocyte injury induced by AQP4-IgG, the authors established a model of astrocyte injury induced by NMO-IgG, and found that NMO-IgG treatment significantly decreased AQP4 levels on astrocyte cell membrane, meanwhile increased CCL2 levels, suggesting CCL2 might be associated with NMO-IgG-induced astrocyte injury.

The authors further used CCL2 small interfering RNA (siRNA) to silence CCL2, and found that CCL2 silence effectively reduced NMO-IgG treatment-induced astrocytes damage. Meanwhile, the authors used CCL2 shRNA adenovirus to selectively knockdown the CCL2 gene in mouse brain astrocytes, followed by NMO-IgG damage. They found that knockdown of CCL2 effectively reduced the lesion volume in NMO mice, suggesting prevention of astrocytic CCL2 significantly reduced NMO-IgG-induced brain damage *in vivo*. In addition, the authors found that NMO-IgG treatment affected a variety of inflammatory signaling pathways in astrocytes. Blocking NF-κB signaling pathway effectively downregulated the expression of inflammatory cytokines. Inhibition of the MAPK p38 signaling pathway also significantly downregulated the expression of CCL2 and CXCL1. Together, the authors found a positive feedback cascade loop of CCL2 and CCR2^+^ to astrocyte injury and the pathogenesis of NMOSD.

## Discussion

CCL2 is a well studied small molecular cytokine of the CC chemokine family. CCL2 has chemotactic activity towards monocytes and is one of the key chemokines regulating the migration and infiltration of monocytes/macrophages, which plays an important role in many diseases such as cancer, autoimmune diseases, neurological diseases, etc. In the autoimmune disease NMOSD, AQP4-IgG is a highly specific diagnostic marker with a specificity of 90%, but its causative factors are unknown. In addition, B cells affect the occurrence of NMOSD by secreting AQP4 antibody and producing cytokines, and monocytes may also participate in the formation of NMOSD. In addition to IL-6 and CCL2, some other cytokines/chemokines were also involved in NMOSD, in which IL-17A, IL-16 and CCL19 acted as proinflammatory cytokines/chemokines in NMOSD pathogenesis. Interestingly, another cytokine IL-19 played a protective role (1).

Currently, the treatments of NMOSD rely on traditional immunosuppressants and modulators, such as Eculizumab, Inebilizumab, and Satralizumab. Satelizumab is an interleukin-6 receptor (IL-6R) inhibitor, which disrupts the JAK/STAT and/or SHP2-MAPK signaling pathways and alleviates IL-6-associated NMOSD injury (12). It has been reported that CCL2 deficiency caused a decrease in marginal zone (MZ) B cells and an increase in germinal center (GC) B cells by up-regulating phosphorylation of the MST1-mTORC1-STAT1 axis (13). Meanwhile, human astrocytes treated with recombinant autoantibodies (Ab) against AQP4 produced a large amount of CCL2, which enhanced the effective recruitment of monocytes. Mitochondrial DNA (mtDNA) could release from astrocytes treated with anti-AQP4 Ab, which further activated monocytes via Toll-like receptor 9 (TLR9). Moreover, mtDNA also enhanced the production of CCL2 in monocytes (14). Importantly, CSF-CCL2 was significantly upregulated in AQP4-IgG seropositive patients, suggesting CCL2 may also facilitate the development of therapeutic drugs for NMOSD.

Generally, the expression of CCL2 was regulated by classical NF-κB signaling and noncanonical pathway. In astrocytes after axonal injury, NF-κB signaling regulated the expression of STAT2 and CCL2, suggesting a central role of astrocytes in guiding immune-glial interactions in the CNS injury response (15). In addition, the expression of CCL2 has been found to be independent on NF-κB in cancer cells. Forkhead box K1 (FOXK1) has been shown to directly link mTORC1 signaling and regulate CCL2 expression, which is independently of NF-κB signaling (16). However, the underlying regulatory mechanism of CCL2 in NMOSD still needed to be studied.

In summary, the study of Wang et al. showed that inhibition of NMO-IgG-induced upregulation of CCL2 could maintain environmental homeostasis, reduce inflammation, and alleviate the deterioration of NMO lesions, suggesting CCL2 may be a promising candidate target for the NMOSD therapy. However, there are several issues worth exploring in the future. First, the binding of NMO-IgG to AQP4 triggers the release of CCL2 by astrocytes, but how the changes of CCL2 in microglia and monocytes, and how the interactions among monocytes, microglia, and astrocytes are still unclear. Second, how cytokine CCL2 regulates AQP4 expression and astrocyte functions are also unknown. Finally, the effects of neutralizing antibodies of CCL2 or targeting drugs in the clinical treatment of NMOSD disease are needed to be validated.

## Author contributions

JZ: Writing – original draft. JC: Conceptualization, Supervision, Writing – review & editing.
